# Metabolic Effect of Dietary Taurine Supplementation on Grouper (*Epinephelus coioides*): A ^1^H-NMR-Based Metabolomics Study

**DOI:** 10.3390/molecules24122253

**Published:** 2019-06-17

**Authors:** Guiping Shen, Shenghao Wang, Jiyang Dong, Jianghua Feng, Jingjing Xu, Feng Xia, Xuexi Wang, Jidan Ye

**Affiliations:** 1 Department of Electronic Science, Fujian Provincial Key Laboratory of Plasma and Magnetic Resonance, Xiamen University, 422 Siming South Road, Xiamen 361005, China; wangshenghaodtc@163.com (S.W.); jydong@xmu.edu.cn (J.D.); jianghua.feng@xmu.edu.cn (J.F.); jingjing@xmu.edu.cn (J.X.); xiafeng@xmu.edu.cn (F.X.); 2 Fisheries College, Xiamen Key Laboratory for Feed Quality Testing and Safety Evaluation, Jimei University, 43 Yindou Road, Xiamen 361021, China; Wangxuexi1993@foxmail.com

**Keywords:** metabolomics, nuclear magnetic resonance, grouper, multivariate statistics, taurine

## Abstract

Taurine is an indispensable amino acid for many fish species and taurine supplementation is needed when plant-based diets are used as the primary protein source for these species. However, there is limited information available to understand the physiological or metabolic effects of taurine on fish. In this study, ^1^H nuclear magnetic resonance (NMR)-based metabolomic analysis was conducted to identify the metabolic profile change in the fish intestine with the aim to assess the effect of dietary taurine supplementation on the physiological and metabolomic variation of fish, and reveal the possible mechanism of taurine’s metabolic effect. Grouper (*Epinephelus coioides*) were divided into four groups and fed diets containing 0.0%, 0.5%, 1.0%, and 1.5% taurine supplementation for 84 days. After extraction using aqueous and organic solvents, 25 significant taurine-induced metabolic changes were identified. These metabolic changes in grouper intestine were characterized by differences in carbohydrate, amino acid, lipid and nucleotide. The results reflected both the physiological state and growth of the fish, and indicated that taurine supplementation significantly affects the metabolome of fish, improves energy utilization and amino acid uptake, promotes protein, lipid and purine synthesis, and accelerates fish growth.

## 1. Introduction

The recent increased expansion of global aquaculture production has led to a greater need for protein sources in feed production [1,2,3]. Because it is a quality protein source, the price of fish meal (FM) has been inflated, causing a sharp increase in the price of commercial aquafeed [1]. Reducing the dietary FM content by using alternative plant protein (PP) sources is required for the sustainable development of the aquaculture industry [2,3]. However, studies have shown that the replacement of FM feed, either partially or completely, with PP sources resulted in inferior fish growth performance together with physiological abnormalities particularly in carnivorous fish [4,5]. This may be due to the lack of certain physiologically active ingredients contained in FM, such as taurine, carnosine, and glutathione [6]. There is thus an urgent requirement for a nutritionally balanced and cost-effective grow-out diet to achieve a desired level of growth [7,8].

Taurine, a sulfonic amino acid, is not present in PP sources but is rich in FM [9]. Many marine larvae and juvenile are unable to synthesize sufficient quantities of taurine and must depend partly on dietary taurine supplementation [4,6]. The efficacy of dietary taurine supplementation for improved growth performance has been reported in Senegalese sole (Solea senegalensis) [3], Nile tilapia (*Oreochromis niloticus*) [6,8,10], barramundi (*Lates calcarifer*) [11] and grouper (*Epinephelus coioides*) [4,7] fed low FM inclusion diets or PP source diets. From the results of these studies, the optimum dietary taurine level for growth of freshwater fish and marine fish were less than 0.5% and more than 1%, respectively. Our previous study further found that the optimum supplementation of taurine was dependent on not only fish species but fish size and feed period duration [10]. Thus, it is necessary to reveal the physiological or metabolic effects of taurine supplementation on fish growth.

Grouper (*Epinephelus coioides*), a carnivorous fish species, is an economically important aquaculture fish species in the world [12]. Taurine has been suggested to be a conditionally essential nutrient in PP-based diets for some carnivorous fish species. In recent years, many studies have focused on the effect of taurine on the growth, nutrient digestibility, and feed utilization of grouper [4,7,13]. However, the underlying physiological or metabolic mechanisms of the effect of taurine supplementation on grouper are not clearly understood [9]. The intestine, being a multifunctional organ central to nutrient uptake and absorption, is thus highly responsive to changes in fish feed [14,15]. Therefore, it constitutes an effective organ for further exploring the metabolic effects of taurine supplementation on fish growth.

Metabolomics is a feasible and promising approach that applies advanced separation and detection methods to investigate the global metabolite change and obtain the related biochemical pathways to elucidate specific sites of perturbations [16,17], and NMR-based metabolomics has been widely applied in various disciplines, including food [18], dietary, and aquaculture studies [10,19,20]. In this study, an untargeted ^1^H-NMR-based metabolomics study of the intestine was used to characterize and compare the metabolic profiles of healthy groupers fed diets supplemented with four different levels of taurine. The aim of this study was to evaluate the effects of dietary taurine supplementation in grouper in terms of fish growth, nutritional digestion, absorption, and transformation on metabolomic.

## 2. Results

### 2.1. Growth of Grouper under Taurine Supplementation

**Figure 1:**
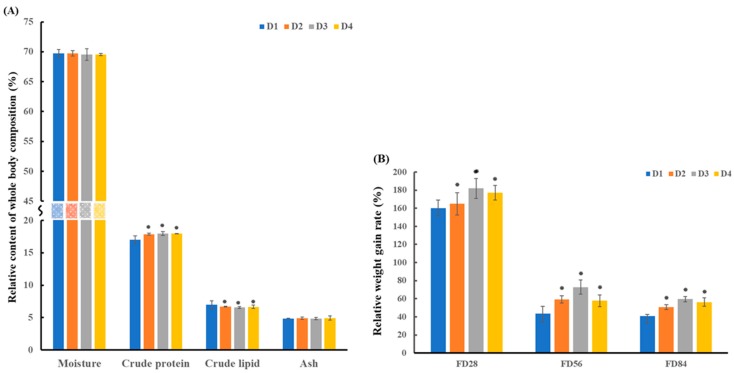
(**A**) The grouper whole-body composition at FD84 and (**B**) the weight gain rate (WGR) at FD28, FD56 and FD84 (**B**) with diets containing different taurine contents (D1: 0.0%, D1: 0.5%, D2: 1.0%, D3: 1.5%). Asterisk (⁕) indicates significant differences between taurine-supplemented groups (D2, D3, and D4) and the control group (D1): *p* < 0.05.

The present results showed that supplementation of dietary taurine significantly affected the whole-body composition and weight gain rate (WGR) of grouper during the feeding trial (Figure 1). No significant differences were observed in moisture and ash contents among dietary treatments. However, crude protein showed an increasing trend in the taurine-supplemented groups (D2, D3, and D4 group) in comparison to the control group (D1), while crude lipid significantly decreased (Figure 1A). These results indicated that taurine supplementation probably influenced the amino acid, protein metabolism, and lipid metabolism of grouper, and thus affected the growth of the fish. Additionally, the WGR of fish was significantly improved in the taurine-supplemented groups during the feeding trail (Figure 1B). WGR showed the highest values at feed day (FD) 28, but decreased with increasing feed duration. Furthermore, during the 84 feed days, WGR showed an increasing trend with increasing supplemental taurine up to 1.0% (D3) and decreased with further taurine supplementation (D4). These results indicate that taurine supplementation indeed facilitated the growth of grouper, and the growth of juvenile fish improved most significantly at FD28.

### 2.2. Metabolic Profiles of the Grouper Intestine

**Figure 2:**
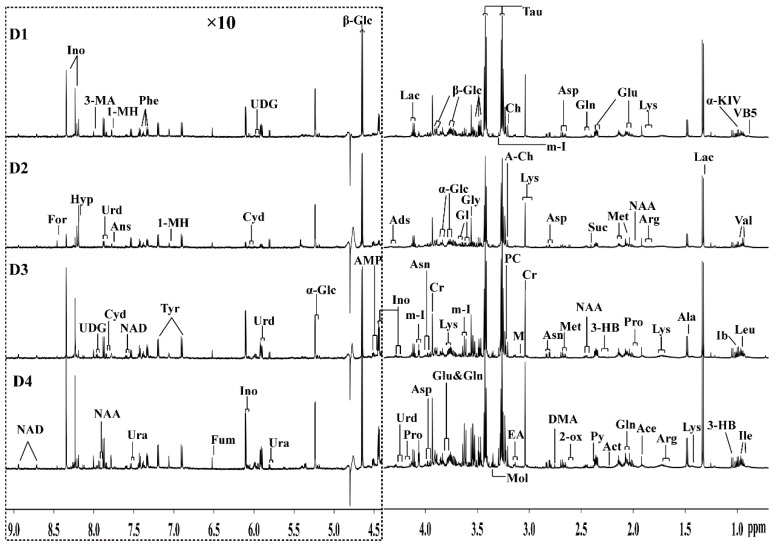
Representative 600 MHz water-suppressed ^1^H-NMR spectra (δ0.5–9.2) of the intestine of grouper fed diets containing different taurine contents (D1: 0.0%, D2: 0.5%, D3: 1.0%, D4: 1.5%) at FD84. The region of δ 4.4–9.2 was vertically magnified 10 times compared with the region δ 0.5–4.4 for the purpose of clarity. Keys:1-MH, 1-methylhistidine; 2-Ox, 2-oxoisocaproate; 3-HB, 3-hydroxybutyrate; 3-MA, 3-methylxanthine; Ace, acetate; A-Ch, acetylcholine; AMP, adenosine monophosphate; Ads, adenosine; Ala, alanine; Ans, anserine; Arg, arginine; Asn, asparagine; Asp, aspartate; Ch, choline; Cyd, cytidine; Cr, creatine; DMA, dimethylamine; ETA, ethanolamine; For, formate; Fum, fumarate; Glu, glutamate; Gln, glutamine; Gl, glycerol; Hyp, hypoxanthine; Ino, inosine; Ib, isobutyrate; Ile, isoleucine; Lac, lactate; Leu, leucine; Lys, lysine; M, malonate; Mol, methanol; Met, methionine; m-I, myo-inositol; NAA, N-acetylaspartate; NAD, nicotinamide adenine dinucleotide; VB5, pantothenate; Phe, phenylanlanine; Pro, proline; Py, pyruvate; Suc, succinate; Tau, taurine; TMA, trimethylamine; Tyr, tyrosine; U, unknown; Ura, urcail; UDG, uridine diphosphate glucose; Urd, uridine; Val, valine; α-Glc, α-glucose; α-KIV, α-ketoisovalerate; β-Glc, β-glucose.

Representative 600 MHz ^1^H-NMR spectra of grouper intestine in all four groups (D1, D2, D3, and D4) fed diets containing four different contents of taurine at FD84 are shown in Figure 2. The peaks in the NMR spectra were assigned and marked according to the published literature [19,20], and confirmed by public NMR databases [21]. A total of 50 metabolites were identified from the ^1^H NMR spectra and the detailed spectral information was listed in Table S2 and the relative concentration value (Mean ± SD) of different metabolites by the calculation of characteristic peaks during the 84 days feeding trail was shown in Table S3. The NMR spectra could intuitively reflect the metabolic changes in the taurine-supplemented groups, such as the high concentrations of taurine, and the low levels of α/β-glucose, lactate, glycine, creatine, and inosine as compared to the control group.

### 2.3. Metabolic Trajectory of Grouper Intestine During the Feed Period

**Figure 3:**
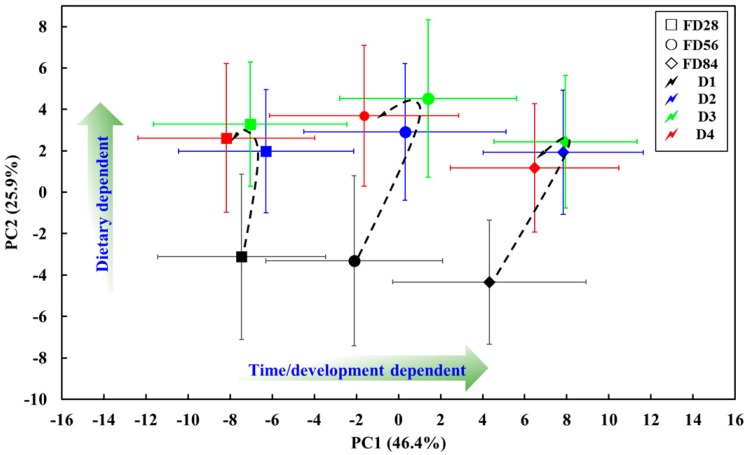
PCA score plots of the intestine of grouper fed four different diets of D1: 0.0% (black), D2: 0.5% (blue), D3: 1.0% (green), and D4: 1.5% (red) at FD28 (☐), FD56 (○) and FD84 (◊). The point with error bars represents the mean and standard deviation of the mean for each diet at different sampling time point, respectively. The hand-drawn trajectories display the path of the metabolic profiles in response to taurine supplementation.

A global principal component analysis (PCA) was performed to investigate the overall metabolic trajectory during the feed periods and to identify the possible outliers between the taurine-supplemented groups and the control group (Figure 3).

As shown in Figure 3, time/development- and dietary-dependent changes were observed along PC1 and PC2, respectively. PC1 and PC2 explained 46.4% and 25.9% of the total variance of the data, respectively. A certain overlap between the clusters from the taurine-supplemented groups (D2 to D4, according to the standard deviation bar of data point) was easily noted during the feed period, indicating that the metabolomic profiles of the taurine-supplemented groups in the intestinal metabolomes were partly similar, and they also demonstrated a slight overlap with the control group (D1) at the FD28 and FD56. However, their obvious separation from D1 at FD84 implied a different metabolic phenotype between D1 and the taurine-supplemented groups, but a slight overlap within the taurine-supplemented groups remained. The changes in the taurine-supplemented groups during the feed period exhibited the same trend, namely a rise between FD28 and FD56 and slight decline at FD84. However, the control group displayed a continuous downtrend from FD28 to FD84. Furthermore, the trajectories of the different diets exhibited the same rising trend from D1 to D3, and down at D4 along the dietary-dependent direction (PC2) during the feed period, and D3 was always distant from D1 on PC1 and PC2. Consequently, this PCA trajectory plot indicates that significant and uneven physiological changes in grouper intestines were induced by taurine supplementation, and the effect of exogenous taurine on group metabolites differed at the different feed periods when comparing with the control group, and the metabolic changes in intestinal tissue in the taurine-supplemented groups were more significant than that of control group at FD56 and FD84. Thus, the metabolomic changes between the taurine-supplemented groups and the control group at FD84 would be further analyzed in the following section.

### 2.4. Physiological and Metabolic Variations in Response to Different Taurine Contents

**Figure 4:**
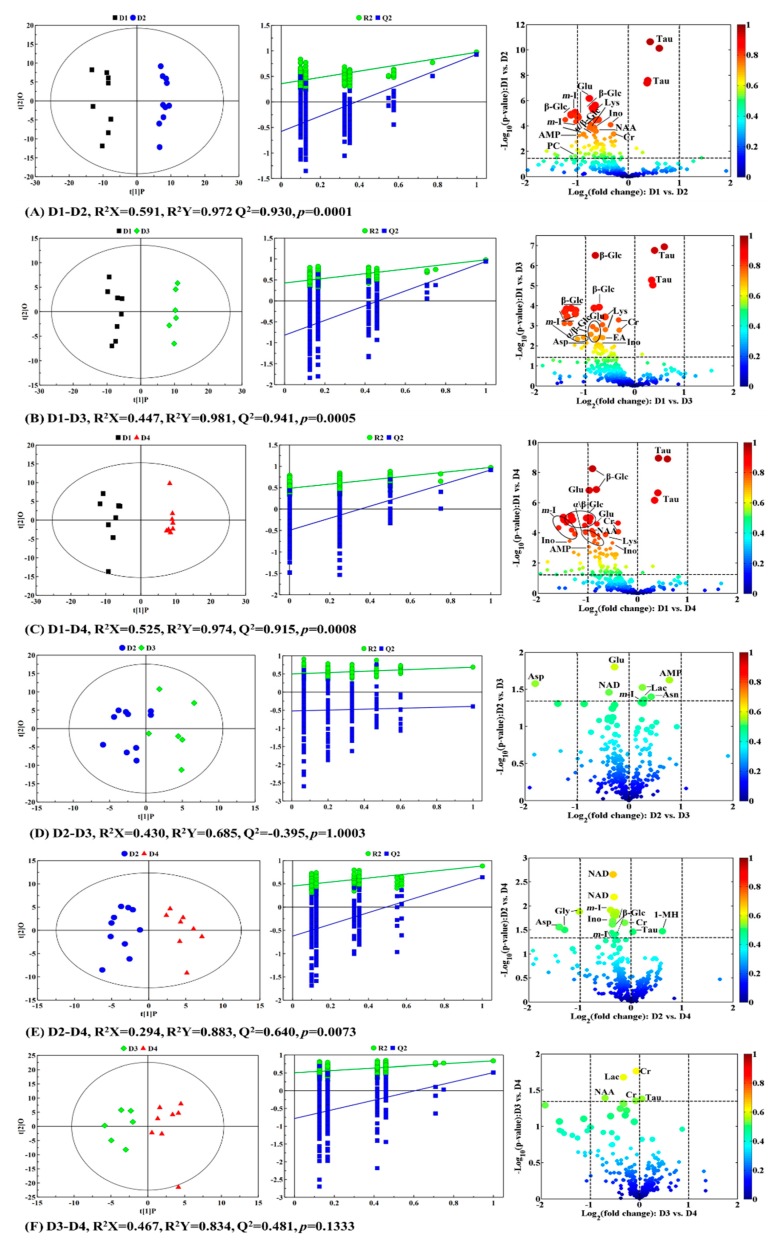
OPLS-DA scores plots (left panel), validation model plots (middle panel) by permutation tests (*n* = 200), and corresponding volcano plots (right panel) derived from the ^1^H-NMR spectra of the intestines of grouper fed diets containing different taurine contents at FD84. (**A**) D1-D2; (**B**) D1-D3; (**C**) D1-D4; (**D**) D2-D3; (**E**) D2-D4; (**F**) D3-D4. The big, middle and small points indicate the variable importance for the projection (VIP) value of the first 5%, second 5%, and the remaining 90%. Keys to the assignments are shown in Figure 2.

In order to evaluate the metabolic information between the different taurine treatment groups, pairwise-comparison between the taurine-supplemented groups and the control group were carried out using partial least squares discriminant analysis (PLS-DA) (Figure S1 in the Supplementary Materials) and orthogonal partial least squares discriminant analysis (OPLS-DA). Volcano plots were used to identify the specific differential metabolites that contributed to the inter-group separation. As shown in the OPLS-DA score plots (left panels in Figure 4A–C,E), the intestinal samples in the different taurine-supplementation groups separated well with a reasonable Q^2^ value (here is greater than 0.640), indicating good predictability of the models and the reliable subsequent analysis of the metabolites. However, the models failed the validation process based on the permutation test at different pairwise-comparison of D2-D3 and D3-D4 (middle panels in Figure 4D,F)), suggesting that similar metabolic profiles existed between these two comparison groups. This was also confirmed by the R^2^Y and Q^2^ values of the models in the pairwise-comparison between the taurine-supplemented groups (D2, D3, and D4) and the control group. As shown in Figure 4, the R^2^Y and Q^2^ values in the pairwise-comparisons of D1-D2, D1-D3, and D1-D4 did not increase with the increase in taurine supplement content, but were highest in D1-D3. However, the values of R^2^Y and Q^2^ increased with the increase in taurine supplement content in the pairwise-comparisons among the taurine-supplemented groups, and the greatest value was observed in D2-D4. The OPLS-DA validation plots from the permutation test also displayed similar trends (middle panels in Figure 4), where the steeper the regression line, the better the NMR data fits the model for R^2^Y and the more significant the metabolic differences. These results imply that taurine supplementation leads to significant metabolic changes, and this effect is most obvious in the D3 group in comparison to the control group.

The differential metabolites, which were determined by *p* < 0.05, |*r*| > 0.5, and VIP values above the top 10%, are visually displayed in volcano plots (above the dashed line in the right panel of Figure 4) and summarized with the evaluation parameters of the models including R^2^X, R^2^Y, Q^2,^ and the *p*-value of the pairwise-comparisons groups in Table 1.

The metabolites changes in grouper intestine can be summarized as follows: (1) A gradual decrease in glucose level (α-/β-glucose) was observed in the D2, D3, and D4 groups as compared with D1, but was inconspicuous in the other pairwise-comparisons of D2-D3 and D3-D4; (2) the level of lactate increased slightly in D3 relative to D2, but decreased in D3 compared with D4. However, nicotinamide adenine dinucleotide (NAD) decreased slightly with the increase in taurine content; (3) a relative increase in taurine in all the taurine-supplemented groups was observed in comparison to D1. However, in all the pairwise-comparisons between the taurine-supplemented groups, taurine only increased obviously in the comparison group of D2-D4; (4) a decline in glutamate, lysine, N-acetylaspartate (NAA), and glycine occurred in the taurine-supplemented groups. Additionally, other amino acids, such as aspartate and proline, decreased slightly only in D3 when compared with D1, but glutamine decreased slightly in D2 and D3. Moreover, a relative increase in the content of asparagine only appeared in D3 as compared with D2, and 1-methylhistidine increased significantly in D4 as compared with D2; (5) a general decrease in creatine and inosine was noted alongside the increase in taurine. Such changes were not so obvious in the pairwise-comparisons of D2-D3 and D3-D4; (6) the level of phosphocholine, choline, *myo*-inositol, and glycerol decreased in all taurine-supplemented groups in comparison to the control group D1. Conversely, the changes in these metabolites, except the *myo*-inositol, were not obvious in the comparative pair-wise analysis between the taurine-supplemented groups, such as D2-D3, D2-D4, and D3-D4. Furthermore, ethanolamine only decreased in D3 as compared with D1; (7) other metabolites, such as adenosine monophosphate (AMP), formate, uridine and fumarate also demonstrated a general decrease with taurine in the D2, D3 and D4. However, AMP also increased in D3 relative to D2. These results indicate that taurine supplementation led to significant metabolome variations, particularly in the D3 group.

**Figure 5:**
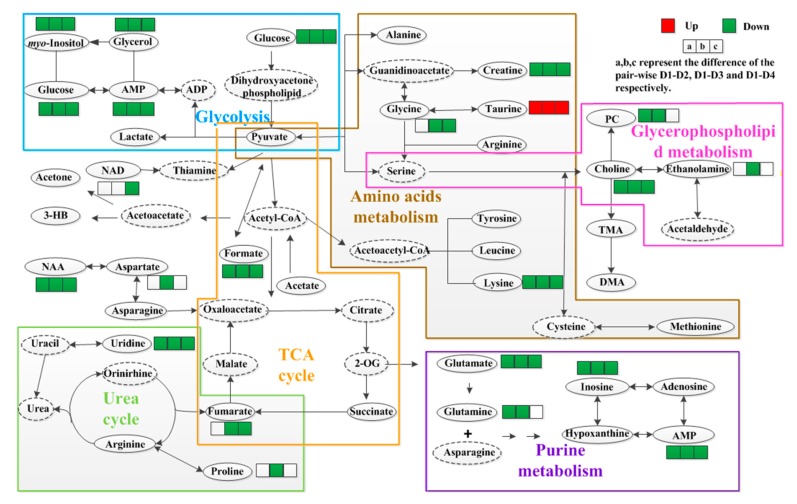
Metabolic pathways affected by dietary taurine in grouper intestinal extracts. Metabolites with a solid oval box were the identified metabolites in the grouper intestinal samples, and those with dashed ellipses were the possible intermediates in metabolic process.

According to the Kyoto Encyclopedia of Genes and Genomes (KEGG) pathway analysis, a number of discriminant metabolic pathways between the taurine-supplemented groups (D2, D3, and D4) and the control group (D1) were identified (Figure 5), including carbohydrate metabolism and glycolysis/gluconeogenesis, amino acids metabolism, protein digestion and absorption, glycerophospholipid metabolism, adenosine triphosphate (ATP)-binding cassette transporters (ABC transporters), tricarboxylic acid (TCA) cycle, urea cycle, choline metabolism, and purine metabolism.

## 3. Discussion

In our previous study [10], the muscle of Nile tilapia was focused in order to explore the metabolic effects of taurine supplementation, in terms of muscle quality, nutrient utilization, and fish growth, due to its widely daily consumption and nutritional value in southern China. In the present study, based on our previous study, we further extended our research to grouper (*Epinephelus coioides*), an economically important aquaculture fish. The intestine, being a multifunctional organ central to nutrient uptake and absorption, is highly responsive to changes in fish feed. Thus, fish intestine constitutes an effective organ for exploring the metabolic effects of taurine supplementation on fish growth. Therefore, an untargeted NMR-based metabolomics technique was used to investigate the effect of taurine on metabolome variations of grouper intestine, and further obtain the information of fish growth, nutritional digestion, absorption, and transformation. The results indicated that dietary taurine supplementation improves energy utilization and amino acid uptake, accelerates protein, lipid, and purine synthesis, and promotes fish growth. These changes reflected the metabolic regulation of grouper intestine tissue by taurine and would help to elucidate the metabolic mechanism of taurine in regulating fish growth.

### 3.1. Energy Metabolism

As shown in Table 1, obvious decreases in α-glucose, β-glucose, AMP, glycerol, *myo*-inositol, and glycine (except in D2) were found in the taurine-supplemented groups relative to the control group. In fact, these metabolites are also involved in the glycolysis and gluconeogenesis pathways (Figure 5) [22]. The significant decrease in α- and β-glucose in the taurine-supplemented groups suggested an intensive oxygenolysis of glucose or conversion to amino acids or other intermediates via pyruvate in the TCA cycle (Figure 5) [21]. This process implies that the protein synthesis would be accelerated due to the acceleration of glycolysis. Therefore, a possible promotion of fat digestion and protein synthesis in the body could be responsible for this process, which corroborates increase in crude protein and the slight decrease in crude lipid content in the grouper body (Figure 1A). In this study, the increase in taurine could be due to the exogenous taurine supplementation in daily diet. Taurine is a conditionally essential amino acid for fish growth and acts synergistically with insulin or insulin-like substances to further promote the utilization of glucose and amino acid uptake in cells [6]. The increase in taurine might accelerate the glycolysis and gluconeogenesis to meet the increased demands of the fish body growth [23]. Moreover, the decrease of α- and β- glucose was accompanied by the significant changes in *myo*-inositol, glycerol and AMP, and was possibly due to the rapid growth of the fish at early and later growth stages (i.e., FD28 and FD84) [24]. Furthermore, metabolites that are related to the energy metabolism exhibited the most obvious decrease in the D3 group. These changes were also verified by the high WGR values of the fish that were fed the taurine-supplemented daily diet, particularly in the period of 1 to 28 d with D3 diet (Figure 1B). The change in energy metabolism might suggest that the taurine supplementation increased the energy consumption, and this is most likely due to the enhanced metabolism (such as protein syntheses and lipid metabolism), which promoted fish growth. This process of optimizing energy utilization leads to the acceleration of growth rates through taurine supplementation in dietary diets as reported in larval Nile tilapia and red sea bream [8,25].

### 3.2. Amino Acid Metabolism

The following amino acids (aspartate, asparagine, glutamate, glutamine, lysine, proline, glycine, and NAA) were significantly decreased compared with the control group. Additionally, lower levels of AMP, NAD, creatine, and inosine were also identified in the taurine-supplemented groups. All these metabolites are correlated with ABC transporters and protein digestion and absorption [21]. Combined with the other metabolic variations in glucose and *myo*-inositol (Figure 5), fish are able to utilize the ATP to transport a wide variety of substances across extra- and intracellular metabolic products, including lipids and sterols, in order to maintain a continuous supply of nourishment for the fish growth [25].

The significantly decreased concentrations of aspartate and asparagine in the grouper intestine implied either a reduced supply of these metabolites or an elevated consumption of them during the development process. In addition, aspartate is an acidic amino acid that is commonly involved in the biosynthesis of glucose, purine nucleotides, pyrimidine bases, and some amino acids, such as lysine, threonine, isoleucine, and methionine as a synthetic precursor [26]. Hence, aspartate can serve as an important energy substrate for fish. Previous studies have also suggested that the high level of taurine in the daily diet of fish could help to increase the activity of aspartate amino transferase (AST) [4]. This could partly explain the significant decrease in aspartate in the pairwise comparisons of D1-D3, D1-D4, and D2-D4 (i.e., obvious decrease in aspartate present in the great dietary taurine-gradient groups). In other words, higher levels of taurine supplementation in the daily diet resulted in more obvious decrease in aspartate. Moreover, the increase in AST activity would promote the metabolism of amino acids and proteins [26], which explains the decreases in the related amino acids. Furthermore, lower aspartate concentrations in the grouper intestine are suggestive of greater dietary amino acid intake demand by the grouper and a higher speed of protein digestion and absorption. Therefore, the significant decrease in aspartate might be a natural stress response during grouper development, and could indicate that grouper development increases when supplemental taurine is provided in the daily diet. In general, the amino acid variations were observed in grouper in response to high dietary taurine supplementation particularly in the D3 group [8], which is in line with the significantly increased growth of fish fed this diet. This is also supported by the results shown in Figure 1.

### 3.3. Purine Metabolism

With respect to purine metabolism, the significant decreases in inosine, glutamine, and AMP could be explained as follows. Inosine is a purine nucleoside that has a hypoxanthine linked by its N9 nitrogen to the C1 carbon of ribose, and hypoxanthine can be converted into AMP, and then to adenosine diphosphate (ADP) and ATP [22]. Inosine is an intermediate in the degradation of purines and purine nucleosides to uric acid and is involved in purine salvage pathways. The decrease in inosine implied the stimulation of DNA synthesis or perhaps a good growth performance of the fish [27]. Furthermore, glutamate serves as the precursor for the synthesis of glutamine, which is involved in multiple metabolic pathways. As shown in Figure 5, the decrease in the glutamine indicated a lower concentration of glutamate, and would lead to a decrease in AMP, which support the gluconeogenic inhibition [28]. The decreased in glutamine, AMP, and some amino acids would promote the oxidation of glucose in order to maintain basic metabolism and the protein synthesis [29]. Therefore, AMP, glutamine and inosine would be converted into energy to support fish growth [20].

### 3.4. Lipid and Glycerophospholipid Metabolism

The obvious decrease in some metabolites in the taurine-supplemented groups compared with the control group could be related to glycerophospholipid metabolism, including that of choline, phosphorylcholine, glycerolphosphocholine, glycerol, inositol, and ethanolamine. First, it should be pointed out that the choline signals indicated as phosphocholine/glycerophosphocholine in Figure 2 are actually mainly from the glycerophosphocholine present in high concentrations in lipoprotein particles [30]. The decreased choline, phosphorylcholine, glycerolphosphocholine, and ethanolamine in the taurine-supplemented groups probably indicated a slowdown of the lipid metabolism, which might indicate that the fat storage of grouper slows as it approaches adulthood. This also corroborates the report of Koven et al., in which taurine appeared to have little effect on lipid storage in the muscle of grouper [4]. Second, the observed low levels of important methyl donors (choline) signify the discontinuation of macromolecular generation in the taurine-supplemented groups [28]. This implied that body mass production was activated and actually promoted in the taurine-supplemented groups. This could be confirmed by the promotion of the growth performance of grouper with the parameters of WGR in response to taurine supplementation at different feeding periods (Figure 1B). Third, glycerol is an important component of triglycerides and phospholipids, which are three-carbon substances that form the backbone of fatty acids in fats. The glycerol component could be converted to glucose by the liver, thereby providing energy for cellular metabolism [26,31]. The lower level of glycerol in the taurine-supplemented groups indicated inhibition of the conversion of glycerol and fatty acids into glucose and the promotion of the body fat accumulation. In other words, the fish adjusts its blood glucose level to meet the requirements of the body through a variety of glucose regulation mechanisms [22,24]. This suggests that the fish could obtain sufficient energy from their daily diet and the regulatory mechanisms associated with increased lipolysis. Furthermore, taurine exerted more significant effects on amino acid metabolism than lipid metabolism during the growth process [26]. In general, high levels of taurine in the daily diet increased the levels of some metabolites related to glycerophospholipid metabolism, leading to the inhibition of proteolysis, which was represented as a decrease in amino acids and non-significant changes in lipids in the intestine during the feed period [29], this explains the increase in crude protein and decrease in crude lipids (Figure 1A).

## 4. Materials and Methods

### 4.1. Diet Preparation

A basal experimental diet was prepared using casein and gelatin as a protein source, fish oil, soybean oil, and soy lecithin as a lipid source and wheat flour as a carbohydrate source. Taurine (food-grade, Beijing Hui Kang Yuan biological technology Co., Ltd., Beijing, China) was incrementally added to the basal diet in ratios of 0.0%, 0.5%, 1.0%, and 1.5%.

The dry ingredients were first ground using a hammer mill and then weighed and combined into a homogeneous mixture, followed by addition of purified water to form a dough. The dough was pelleted through a 2.5-mm sieve using multifunctional spiral extrusion machinery (CD4XITS, South China University of Technology, Guangzhou, China). The pellets were oven-dried at 60 °C for 24 h and then sealed in plastic bags and kept at −20 °C until feeding. According to the different taurine ratios, four experimental diets were prepared and labeled as D1, D2, D3, and D4, respectively. The ingredients and proximate compositions of the experimental diets are shown in Table S1 in the Supplementary Materials.

### 4.2. Fish Rearing and Experimental Conditions

All experimental procedures and protocols were approved by the local animal ethics committee at Jimei University (JMU-2011-58). The grouper juveniles obtained from a local commercial farm in Xiamen were transported to the aquaculture laboratory at Jimei University. Prior to the start of the experiment, the fish were initially fed with a commercial fish feed and were acclimatized to the rearing conditions in a closed recirculating system consisting of two circular fiber glass tanks (0.85 m height × 1.22 m upper diameter, 1.04 m lower diameter) with a PolyGeyser bead filter (Aquaculture Systems Technologies, LLC., Berkeley, CA, USA) for two weeks.

A total of 400 animals, fifty-six days old with an average initial weight of 13.85 ± 0.25 g (means ± SD), were stocked in a randomized block design with 25 fish per tank (total of 400 groupers in 16 tanks), used to compare growth performance and metabolic profiles (the groupers were placed in four tanks/diet and assigned to four groups as D1 (control), D2, D3, and D4 according to their diets). Each group was divided into four identical 150 L cylindrical tanks (0.6 m in diameter and 0.75 m in height) within a recirculating system connected to a circulation pump, with biological and mechanical filters, and supplemental aeration. The fish in each group were hand-fed one of the diets to satiation at 08:00, 13:00, and 18:00 h daily under a natural photoperiod for 84 days. Half an hour after each feeding, excess feed was collected by siphoning, then dried at 70 °C, and weighed in order to calculate the feed intake. Dissolved oxygen and water temperature were measured daily at 12:00 h and nitrite-N was monitored twice weekly with a multi-parameter photome (HI83200; Hanna Instruments, Woonsocket, RI, USA). During the feeding trial, the water temperature, dissolved oxygen and nitrite-N ranged were 26.4 ± 1.1 °C, 5.8 mg/L ± 0.4 mg/L and 0.127 ± 0.055 mg/L, respectively.

### 4.3. Sampling Procedure

At weeks 4, 8, and 12 of the feeding trial (tapped as FD28, 56, and 84 in the following context) [1,32], three fish were randomly sampled from each tank (12 fish/diet, 48 fish per sampling event) after 12 h of fasting, and anesthetized using an overdose of tricaine methanesulfonate solution (MS 222, Sigma-Aldrich Shanghai Trading Co. Ltd., Shanghai, China, 100 mg/L). The fish were weighed individually and killed by sharp blow to the head. The visceral cavity was then opened, and the intestine was immediately dissected out. A 3-cm length sample from the anterior and middle section of the intestine was selected and washed using deionized water, and any adherent tissue or intestinal contents were removed gently. The intestinal sample was placed into pre-labeled cryovials and flash frozen in liquid nitrogen, and eventually stored at −80 °C.

The fish carcasses were weighed and minced and then dried at 70 °C for whole-body compositional analysis. The fish samples were ground into fine powder using a laboratory grinder. Moisture, crude protein, crude lipid, and ash in these samples were determined according to the methods of the Association of Official Chemists (AOAC) [29]. The fish growth performance of WGR was calculated as follows:

WGR (%) = 100 × (*W*_f_ − *W*_i_)/*W*_i_ (1)

where *W*_f_ (g) and *W*_i_ (g) are the final and initial body weight, respectively. Significant differences between the taurine treatments were calculated using Student’s *t*-test.

Prior to the NMR analysis, the intestine samples were extracted according to a protocol to obtain a freeze-dried powder [33]. In brief, fish intestinal samples (100 mg) were homogenized for 30 s in 400 µL of methanol and 125 µL of deionized water at 4 °C. The homogenates were transferred to a 2.5 mL tube, combined with 400 µL of chloroform and 400 µL of deionized water, and the mixture was vortexed for 60 s. The use and disposal of chloroform and chloroform-contaminated materials were in compliance with Xiamen University laboratory safety management regulations. After 10 min partitioning on ice, the samples were centrifuged for 5 min (10,000× *g*, 4 °C). The upper supernatants were transferred to 1.5 mL tubes, and lyophilized for 24 h to remove methanol, chloroform and water, and then stored at −80 °C until the NMR test.

### 4.4. Sample Preparation and ^1^H-NMR Spectroscopy

The aqueous freeze-dried fish intestine powder was dissolved in 600 µL of 99.9% D_2_O phosphate buffer (0.2 M, pH 7.0) with 0.05 wt.% sodium 3-(trimethylsilyl) propionate-2,2,3,3-d_4_ (TSP). The extracted intestine buffer mixture was kept at room temperature for 5 min, and then centrifuged for 10 min (6000× *g*, 4 °C) to remove suspended debris. The supernatant (550 μL) was then pipetted into a 5-mm NMR tube and stored at 4 °C before analysis by an NMR spectrometer.

All the intestinal samples were analyzed randomly at 298 K by using a Bruker AMX-600 spectrometer (Bruker BioSpin GmbH, Karlsruhe, Germany) equipped with a 5-mm CPBBO CryoProbe operating at 600.13 MHz. 1H-NMR spectra were acquired by a one-dimensional pulse sequence based on a NOESY (nuclear Overhauser effect spectroscopy) pulse sequence (RD-90°-t_1_-90°-t_m_-90°-Acq) with water suppression (NOESYPR1D). The 90° pulse length was adjusted to approximately 12 μs, and 64 transients were collected into 32 K data points for each spectrum with a spectral width of 12 KHz. The acquisition time was 2.65 s, and the relaxation delay was 4.0 s, with a fixed interval *t*_1_ of 4 µs. The water resonance was irradiated during the relaxation delay and the mixing time *t*_m_ was 100 ms.

### 4.5. Preprocessing of NMR Spectra and Multivariate Analysis

All free induction decays (FIDs) were multiplied by an exponential function equivalent to a 0.3 Hz exponential line-broadening factor and extended to 64 K data points by zero padding prior to fast Fourier transformation. All of the ^1^H-NMR spectra were manually phased and baseline-corrected using MestReNova (V7.1.0-9185, Mestrelab Research S.L., Santiago de Compostela, Spain), and referenced to TSP at 0.0 ppm. Spectral regions including residual water resonance (5.22~4.16 ppm), residual methanol (3.37~3.34 ppm), TSP signal (0.0 ppm), and peak-free baseline were removed from the spectra. Then, spectra over the range of 9.2~0.5 ppm were binned into 662 buckets using the adaptive binning method [34]. Each spectrum was normalized using probabilistic quotient normalization (PQN) to account for sample dilution effects, thus, facilitating comparability of the samples [35]. Interference factors, which were independent of the purpose of the study, were filtered out using analysis of variance-principal component analysis (ANOVA-PCA) [36].

The NMR data were imported into SIMCA-P software (version 14.0, Umetrics AB, Umeå, Sweden) for multivariate statistical analysis (raw data is available in the Supplementary Materials). PCA was conducted to give an overview of the data distribution and potential outliers. Then, PLS-DA and OPLS-DA were implemented on the NMR data in order to identify specific metabolomic differences between the taurine-supplemented groups and the control group. Pairwise comparisons were performed and validated with 7-fold cross validation and permutation test (permutation number *n* = 200) in the PLS-DA and OPLS-DA methods. The quality of the model was described by the cross-validation parameter Q^2^, indicating the predictability of the model, and R^2^, indicating the explained variation for the NMR data. An additional validation method, CV-ANOVA was conducted to validate the models. In all instances, the level of statistical significance was set as *p* < 0.05.

In addition, the relative concentrations of the metabolites were compared based on the fold-change by calculating the integral area of the corresponding signals and statistically analyzing with Student’s *t*-test to screen the reliability of the characteristic metabolites. In order to avoid the influences of the spectral overlapping, only those characteristic peaks with least overlapping were chosen for quantitation of the corresponding metabolites. A volcano plot was used to summarize both the *t*-test and fold-change criteria in a single plot, and a typical scatter plot of the −*log*_10_ (*p*-value) against *log*_2_(fold-change) which represented as the y- and x-axis, respectively. The metabolites contributing towards the metabolome differences in the pairwise-comparisons were marked with different circles sizes and were color-coded based on the variable importance for the projection (VIP) and the corresponding absolute correlation coefficients (|*r*|) constructed from the OPLS-DA analysis, respectively. The larger circle size corresponds to a larger VIP value, and a warm color corresponds to a significant difference between classes, while a cool color represents the opposite. The four-dimensional volcano plots thus provide integrated information regarding the metabolomic differences between the different treatment groups. Metabolites exhibiting significant change were determined by combing the restrictions of three dimensions: *p* < 0.05, |*r*| > 0.5 and VIP values above the top 10%, and tended to be located in the upper zones of the plot, segmented by the horizontal threshold line *p* = 0.05, with the larger, middle circle sizes and warmer colors. The volcano plots were generated with MATLAB scripts (downloaded from http://www.mathworks.com) with some in-house modifications.

### 4.6. Metabolic Pathways Analysis

To gain a deeper insight into the effect of exogenous taurine on the differences in metabolic pathways between the taurine-supplemented groups and the control group, a comprehensive pathway analysis via KEGG and the MetaboAnalyst online service (http://www.metaboanalyst.ca/) was conducted on the differential metabolites derived from the different comparison models [37,38].

## 5. Conclusions

In summary, the metabolic changes in grouper intestine reflected both the physiological state and growth of the fish, and indicated that taurine supplementation significantly affects the energy metabolism, amino metabolism, and lipid metabolism of grouper, and accelerates the fish growth, which are consistent with our previous studies on tilapia [10]. Significantly difference with the metabolic changes in tilapia muscle tissue is that taurine also promotes purine synthesis, and indirectly improves energy utilization and amino acid uptake, accelerates the protein and lipid synthesis in grouper. In addition, the taurine benefit was sensitive to changes in supplementation level. The most significant metabolic changes and fish growth were found in 1.0% taurine supplementation (D3) for grouper under these experimental conditions. Our study might provide an important reference for fish feed related diet and nutrition studies and offer guidance on the implementation of fish culture in metabolism. Further in-depth investigations are required to identify the metabolic pathways affected by taurine and to determine how taurine is synthesized and metabolized in fish.

## Figures and Tables

**Table 1:** OPLS-DA coefficients derived from the NMR data of grouper fed diets with different taurine levels at FD84.

Metabolites	D1-D2		D1-D3		D1-D4		D2-D3		D2-D4		D3-D4	
	R^2^X = 0.762 ^a^ R^2^Y = 0.974 Q^2^ = 0.920 *p* = 0.000		0.738 0.979 0.908 0.000		0.807 0.972 0.910 0.000		0.668 0.852 0.209 1.000		0.703 0.949 0.779 0.007		0.763 0.876 0.259 0.1333	
	Fold ^b^	r ^c^	Fold	r	Fold	r	Fold	r	Fold	r	Fold	r
**Energy metabolism**												
α-Glucose	0.628	−0.780	0.603	−0.810	0.573	−0.760			0.865	−0.724		
β-Glucose	0.589	−0.778	0.553	−0.824	0.567	−0.792			0.803	−0.742		
Lactate							1.205	0.500			0.796	−0.560
NAD							0.756	−0.530	0.794	−0.615	0.834	−0.513
**Lipid metabolism**												
Phosphocholine	0.484	−0.524	0.445	−0.546								
Choline	0.646	−0.560	0.492	−0.660	0.591	−0.672						
*myo*-Inositol	0.482	−0.769	0.416	−0.801	0.418	−0.797	1.175	0.502	0.782	−0.508	0.761	−0.520
Glycerol	0.636	−0.677	0.625	−0.669	0.587	−0.742						
Ethanolamine			0.637	−0.717								
**Amino acid metabolism**												
Taurine	1.316	0.947	1.298	0.862	1.359	0.943			1.023	0.499		
Aspartate			0.595	−0.620					0.377	−0.518		
Asparagine							1.339	0.518				
Glutamate	0.667	−0.770	0.694	−0.643	0.575	−0.823	0.823	−0.591				
Glutamine	0.781	−0.551	0.775	−0.524								
Lysine	0.649	−0.858	0.639	−0.867	0.640	−0.813						
Proline			0.696	−0.537								
Glycine			0.573	−0.645	0.517	−0.713			0.451	−0.539		
NAA	0.642	−0.760			0.534	−0.823					0.613	−0.552
1-Methylhistidine									1.529	0.502		
Creatine	0.816	−0.741	0.804	−0.759	0.763	−0.825			0.916	−0.533	0.955	−0.623
Inosine	0.792	−0.697	0.646	−0.595	0.723	−0.708			0.780	−0.540		
Other												
AMP	0.563	−0.719	0.497	−0.737	0.507	−0.750	1.707	0.561				
Formate	0.664	−0.745	0.541	−0.769	0.599	−0.798						
Uridine	0.691	−0.698	0.587	−0.712	0.638	−0.721						
Fumarate			0.723	−0.645	0.726	−0.700						

^a^ The evaluation parameters of the models including R^2^X, R^2^Y, Q^2,^ and *p*-values. ^b^ Fold change values, greater or less than 1 indicates the metabolites that are more or less abundant in the latter than in the former, respectively. ^c^ Correlation coefficients, positive and negative signs indicate positive and negative correlations in the concentrations, respectively.

## Supplementary Materials

The following are available online, Figure S1: PLS-DA scores plots (left panel) and validate model plots (right panel) by permutation tests (*n* = 200) for 1H NMR spectra of grouper intestine between different taurine-supplemented groups at FD84. (A) D1-D2; (B) D1-D3; (C) D1-D4; (D) D2-D3; (E) D2-D4; (F) D3-D4, Table S1: Ingredients and composition of experimental diets (on an as fed basis), Table S2: Metabolites identified from NMR spectra of grouper intestine and the corresponding assignments, Table S3: Relative concentration value (Mean ± SD) of different metabolites by the calculation of characteristic peaks during the 84 days feeding trail. Excel file: Raw data used for multivariate analysis.
