# Supplementary material for: Metabolic Effect of Dietary Taurine Supplementation on Grouper (Epinephelus coioides): A 1H-NMR-Based Metabolomics Study
Source: Molecules. 2019 Jun 17;24(12):2253. doi: 10.3390/molecules24122253 (PMC6630984; doi:10.3390/molecules24122253)
Supplement: Supplementary file 1 [file molecules-24-02253-s001.zip › molecules-521889-2nd revise-SM/molecules-521889-2nd revise-SM.pdf]

Supplementary Materials

Metabolic Effect of Dietary Taurine Supplementation on Grouper (*Epinephelus coioides*): a ^{1}H NMR-based Metabolomics Study

Guiping Shen [1]*, Shenghao Wang [1], Jiyang Dong [1], Jianghua Feng [1], Jingjing Xu [1], Feng Xia [1], Xuexi Wang [2], Jidan Ye [2]*

[1] Department of Electronic Science, Fujian Provincial Key Laboratory of Plasma and Magnetic Resonance, Xiamen University, Xiamen 361005, China; 545926975@qq.com (S.W.); jydong@xmu.edu.cn (J.D.); jianghua.feng@xmu.edu.cn (J.F.); jingjing@xmu.edu.cn (J.X.); xiafeng@xmu.edu.cn (F.X.);

[2] Fisheries College, Xiamen Key Laboratory for Feed Quality Testing and Safety Evaluation, Jimei University, Xiamen 361021, China; 1508498684@qq.com (X.W.)

* Authors to whom correspondence should be addressed:

[1] 422 Siming South Road, Xiamen University, Xiamen, Fujian Province 361005, China. Tel.: +86 592 2184026, Fax: +86 2189426.

Email address: gpshen@xmu.edu.cn (G.S.)

[2] 43 Yindou Road, Jimei Univesity, Xiamen, Fujian Province 361021, China.

Tel./fax: +86 592 6181054.

Email address: 200461000010@jmu.edu.cn (J.Y.)

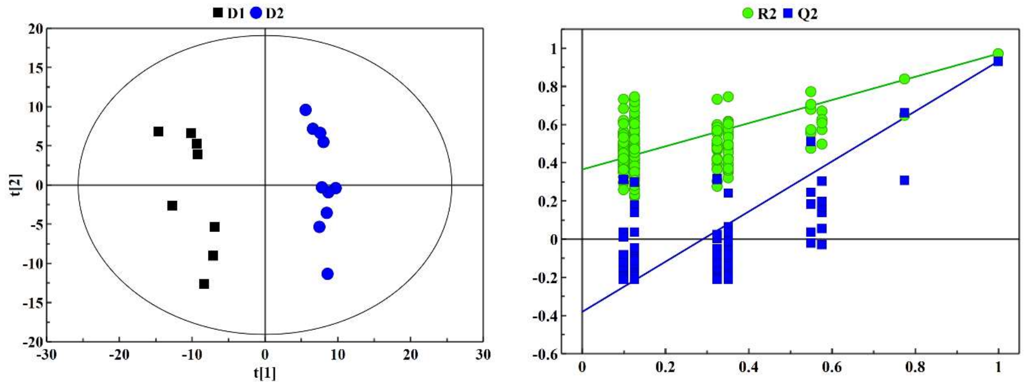

(A) D1-D2, R^2X=0.591, R^2Y=0.972, Q^2=0.932

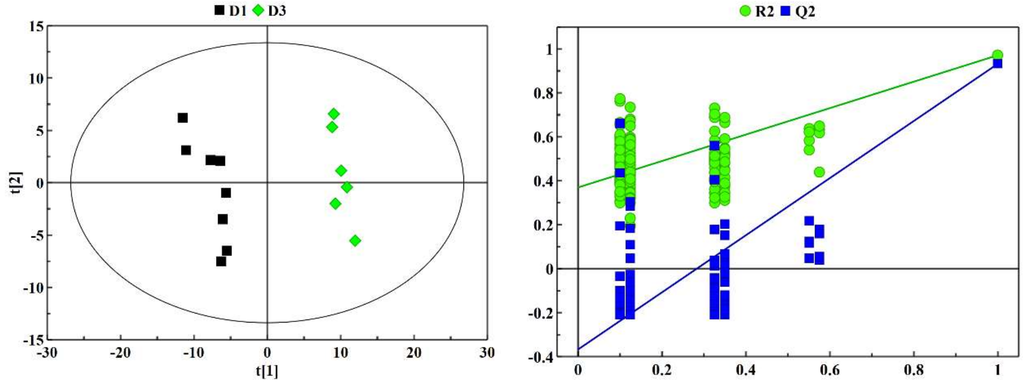

(B) D1-D3, R^2X=0.447, R^2Y=0.981, Q^2=0.875

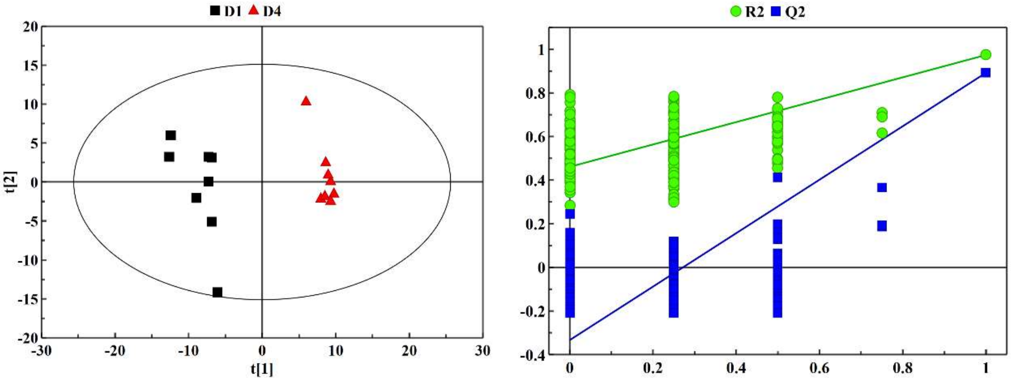

(C) D1-D4, R^2X=0.525, R^2Y=0.974, Q^2=0.892

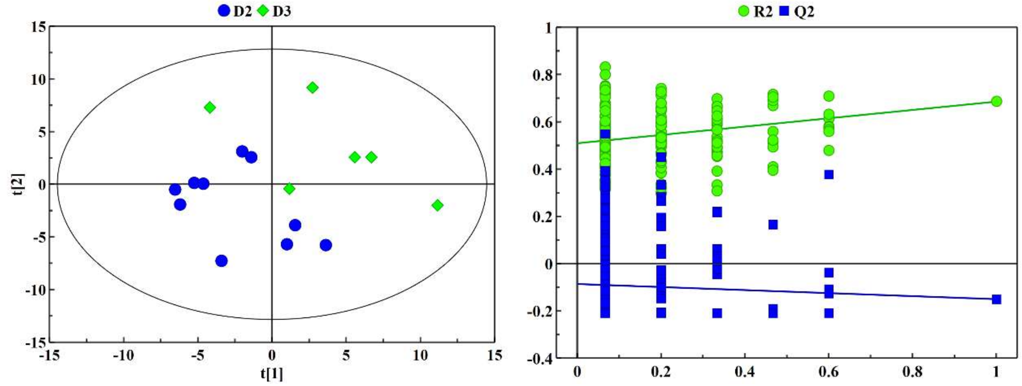

(D) D2-D3, R^2X=0.430, R^2Y=0.685, Q^2=-0.150

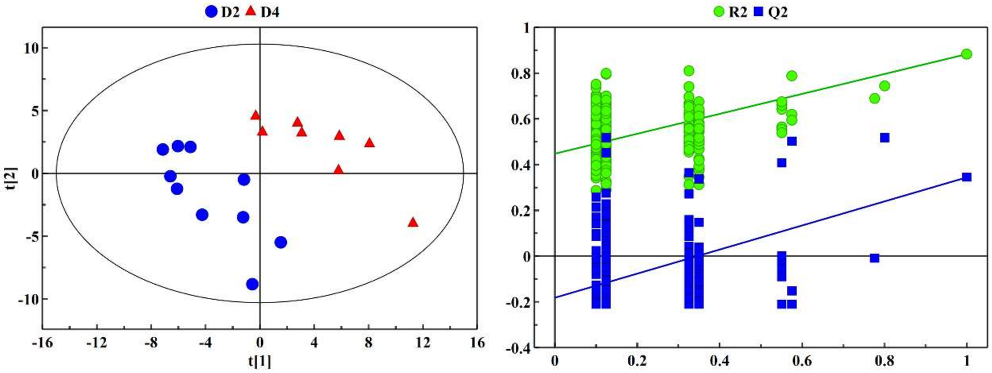

(E) D2-D4, R^2X=0.294, R^2Y=0.883, Q^2=0.344

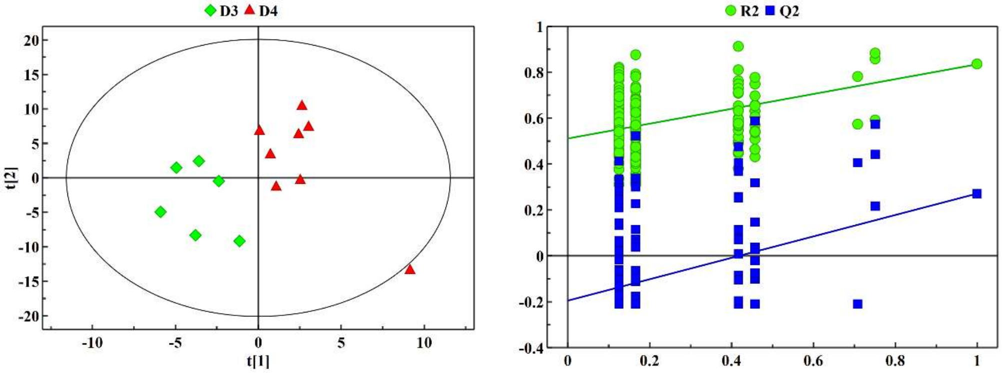

(F) D3-D4, R^2X=0.467, R^2Y=0.834, Q^2=0.271

Figure S1. PLS-DA scores plots (left panel) and validate model plots (right panel) by permutation tests (n=200) for ^{1}H NMR spectra of grouper intestine between different taurine-supplemented groups at FD84. (A) D1-D2; (B) D1-D3; (C) D1-D4; (D) D2-D3; (E) D2-D4; (F) D3-D4.

Table S1 Ingredients and composition of experimental diets (on an as fed basis)

Ingredients (%)	Diets at different taurine inclusion contents			
	D1 (0.0%)	D2 (0.5%)	D3 (1.0%)	D4 (1.5%)
Casein	47	47	47	47
Gelatin	8	8	8	8
Wheat flour	24	24	24	24
Fish oil	3	3	3	3
Soybean oil	3	3	3	3
Soybean lecithin	3	4	4	4
Vitamin mix[1]	0.2	0.2	0.2	0.2
Mineral mix[2]	0.3	0.3	0.3	0.3
Vitamin C	0.02	0.02	0.02	0.02
Monocalcium phosphate	2	2	2	2
Choline chloride	0.3	0.3	0.3	0.3
Shrimp meal	3	3	3	3
Microcrystalline cellulose	3.18	2.68	2.18	1.68
Taurine	0	0.5	1.0	1.5
**Nutrient level (%)**				
Dry matter	90.81	90.76	90.24	90.21
Crude protein	49.55	49.21	49.13	49.56
Crude lipid	8.78	8.61	8.54	8.71
Ash	2.21	2.54	2.35	2.42
Taurine	0.10	0.49	0.96	1.47

[1] Vitamin premix consisted of (mg /kg diet) the following: vitamin A 10; vitamin D 10; vitamin K 40; vitamin E 100; vitamin B1 60; vitamin B2 70; vitamin B6 80; vitamin B12 0.4; nicotinic acid 200; calcium pantothenate 200; biotin 2; inositol 500; folicacid 8.

[2] Mineral premix consisted of (mg /kg diet) the following: $FeSO_4 \cdot 7H_2O$ 497.84, $CuSO_4 \cdot 5H_2O$ 11.8, $ZnSO_4 \cdot 7H_2O$ 175.84, $MnSO_4 \cdot 4H_2O$ 121.8, $CoCl_2 \cdot 6H_2O$ 0.18, KIO_3 0.51, Na_2SeO_3 0.33.

Table S2 Metabolites identified from NMR spectra of grouper intestine and the corresponding assignments

Number	Metabolites	Abbreviation	HMDB ID	^{1}H NMR chemical shift (ppm) (multiplicity) [a]	Assignment [b]
1	1-Methylhistidine	1-MH	0000001	7.06(s), 7.78(s)	CH(2), CH(4)
2	2-oxoisocaproate	2-Ox	0000695	2.61（d）	CH_2
3	3-Hydroxybutyrate	3-HB	0041603	1.20(d), 2.31(dd), 2.40(m), 4.16(m)	CH_3
4	3-Methylhistidine	3-MH	0000479	7.03(s)	CH(4)
5	Acetate	Ace	0029699	1.92(s)	CH_3,
6	Adenosine	Ade	0000050	4.29(dd),4.44(dd),6.11(d), 8.37(s)	CH(2), N-CH=N
7	Adenosine monophosphate	AMP	0000045	4.52(d),6.15(d),8.27(s), 8.61(s)	CH-N, N-CH=N, N-CH'=N
8	Alanine	Ala	0028878	1.48(d)	CH_3
9	Anserine	Ans	0000194	7.78（s）	CH(4)
10	Asparagine	Asn	0029012	2.88(dd),2.95(dd),3.99(dd)	β-CH, β’-CH, α-CH
11	Aspartate	Asp	0028749	2.70(dd),2.81(dd),3.94(dd)	β-CH, β-CH', α-CH
12	Choline	Ch	0000097	3.20(s)	CH_3
13	Creatine	Cr	0000064	3.04(s), 3.93(s)	CH_3, CH_2
14	Cytidine	Cyd	0000089	5.87(d),7.81(d)	CH(5),CH(6)
15	Dimethylamine	DMA	0000087	2.72(s)	CH_3
16	Ethanolamine	EA	0000149	3.11(t),3.86(t)	CH_2NH_2, CH_2OH
17	Formate	For	0031351	8.46(s)	CH
18	Fumarate	Fum	0031257	6.52(s)	CH
19	Glutamate	Glu	0028994	2.08(m), 2.11(m), 2.35(m), 3.75(t)	α-CH, β-CH_2, γ-CH_2
20	Glutamine	Gln	0028861	2.14(m), 2.45(m), 3.75(t)	γ-CH_2, α-CH
21	Glycerol	G	0000131	3.55(m), 3.66(dd), 3.78(m)	CH_2, CH_2'
22	Glycine	Gly	0000123	3.56(s)	CH
23	Hypoxanthine	HX	0000157	8.20(s)	CH(7)
24	Inosine	Ino	0000195	4.26(dd),6.08(d),8.25(s), 8.34(s)	CH(5), CH(2), N-CH'=N
25	Isobutyrate	IB	0037189	1.07(d)	β-CH_3
26	Isoleucine	Ile	0028844	0.94(t), 1.01(d),1.26(m)	δ-CH_3, β-CH_3
27	Lactate	Lac	0037212	1.33(d), 4.11(q)	CH_3, CH
28	Leucine	Leu	0028735	0.96(t)	CH_3
29	Lysine	Lys	0003405	1.46(m), 1.73(m), 1.91(m), 3.03(m), 3.76(t)	δ-CH_3, β-CH_3, ε-CH_3, α-CH_3
30	Malonate	M	0029537	3.11(s)	CH_2
31	Methanol	Mol	0001875	3.36(s)	CH_3
32	Methionine	Met	0002005	2.14(s), 2.63(t)	S-CH_3, β-CH_3, S-CH_2

33	myo-Inositol	m-I	0000213	3.52(dd), 3.61(dd), 4.07(m)	CH(1), CH(2), CH(3), CH(4), CH(6)
34	Nicotinamide adenine dinucleotide	NAD	0000902	4.37(m),4.42(m),4.49(m), 4.51(m),6.04(d),6.12(d), 8.42(d),8.83(d),9.14(d),9.34(s)	CH(2), CH(3), CH-OH, O-CH-N, CH(7), CH_2(39)
35	Phenylalanine	Phe	0028892	4.00(m),7.32(d), 7.37(t), 7.42(s)	o-CH, p-CH, m-CH
36	Phosphocholine	PC	0001565	3.21(s),4.18(m)	CH_3
37	Proline	Pro	0029113	3.33(m)	γ-CH_2, half β-CH_2, half β-CH_2 δ-CH_2, CH
38	Pyruvate	Py	0031643	2.37(s)	CH_3
39	Succinate	Suc	0033837	2.40(s)	CH
40	Taurine	Tau	0000251	3.27(t),3.42(t),	CH_2SO_3, NCH_2
41	Trimethylamine	TMA	0000906	2.89(s)	CH_3
42	Tyrosine	Tyr	0028765	6.90(d), 7.19(d)	m-CH, o-CH
43	Uracil	Uc	0000300	5.80(d),7.55(d)	CH(5), CH(6)
44	Uridine	Ud	0000296	4.24(t),4.36(t),5.91(d), 5.93(d),7.89(d)	CH(a), CH(b), CH(c), CH(5), CH(6)
45	Uridine diphosphate glucose	UDG	0000286	5.61(dd),5.97(m),7.96(d)	CH(2), CH(21), CH(31), N-CH=C
46	Urocanate	Urc	0000301	6.39(d),7.38(s),7.81(s)	CHCOOH, CH=C, CH=N
47	Valine	Val	0000883	0.99(d), 1.04(d),2.28(m)	γ-CH_3, γ-CH_3'
48	α-Glucose	α-Glc	0062621	3.42(t), 3.54, 3.71(t), 3.73(m), 3.84(m), 5.24(d)	CH(2), CH(3), CH(5&6'), CH(1)
49	α-Ketoglutarate	Kg	0061388	2.45(t),3.01(t)	γ-CH_2
50	β-Glucose	β-Glc	0062476	3.24(dd), 3.41(t), 3.46(m), 3.49(t), 3.90(dd)	CH(5), CH(3), CH(6), CH(1)

[a] Multiplicity: s, singlet; d, doublet; t, triplet; q, quartet; dd, doublet of doublets; m, multiplet.

[b] Assignment: according to the HMDB database (www.hmdb.ca).

Table S3 Relative concentration value (Mean ± SD) of different metabolites by the calculation of characteristic peaks during the 84 days feeding trail

Metabolites	characteristic peaks (ppm)	FD28				FD56				FD84			
		D1	D2	D3	D4	D1	D2	D3	D4	D1	D2	D3	D4
1-Methylhistidine	7.06(s)	0.85±0.20	0.76±0.24	1.20±0.41	1.06±0.22	0.82±0.13	1.05±0.25	1.03±0.22	1.24±0.27	0.46±0.21	0.60±0.09	0.63±0.16	0.96±0.21
2-oxoisocaproate	2.61(d)	0.03±0.03	0.06±0.09	0.16±0.18	0.10±0.10	0.02±0.03	0.03±0.05	0.01±0.03	0.03±0.04	0.04±0.06	0.08±0.06	0.11±0.11	0.04±0.02
3-Hydroxybutyrate	1.20(d)	0.24±0.08	0.13±0.04	0.23±0.13	0.95±0.54	0.25±0.15	0.16±0.05	0.24±0.07	0.15±0.03	0.39±0.43	0.23±0.04	0.23±0.09	0.27±0.09
3-Methylhistidine	7.03(s)	0.85±0.20	0.76±0.24	1.20±0.41	1.06±0.22	0.82±0.13	1.05±0.25	1.03±0.22	1.24±0.27	0.46±0.21	0.60±0.09	0.63±0.16	0.75±0.21
Acetate	1.92(s)	1.55±0.64	1.51±0.68	1.05±0.26	1.59±0.54	0.71±0.24	0.97±0.32	0.86±0.27	0.96±0.22	0.90±0.33	1.14±0.24	0.98±0.15	0.96±0.18
Adenosine	8.37(s)	0.56±0.74	0.54±0.66	0.88±0.93	0.68±0.92	1.58±0.73	1.83±0.64	1.75±1.02	1.81±0.91	2.55±0.41	2.79±0.38	2.35±1.28	2.40±0.59
Adenosine monophosphate	8.61(s)	0.06±0.05	0.10±0.06	0.06±0.04	0.09±0.04	0.05±0.04	0.04±0.03	0.06±0.06	0.05±0.04	0.01±0.02	0.01±0.02	0.01±0.01	0.01±0.01
Alanine	1.48(d)	3.21±0.33	2.71±0.44	2.95±0.62	3.59±0.43	2.84±0.55	3.11±0.76	3.19±0.64	3.45±0.67	1.70±0.41	2.07±0.27	2.13±0.28	2.19±0.47
Anserine	7.78(s)	0.59±0.16	0.41±0.22	0.52±0.43	0.64±0.42	0.56±0.22	0.66±0.27	0.67±0.30	0.78±0.33	0.33±0.15	0.33±0.13	0.44±0.22	0.48±0.19
Asparagine	2.95(dd)	0.45±0.33	0.66±0.19	1.08±0.45	0.64±0.35	0.64±0.13	0.76±0.22	0.70±0.24	0.89±0.26	0.39±0.10	0.41±0.16	0.55±0.07	0.49±0.17
Aspartate	2.81(dd)	0.56±0.60	0.23±0.31	0.32±0.46	0.58±0.51	0.22±0.27	0.11±0.21	0.11±0.14	0.10±0.11	1.16±0.39	0.83±0.61	0.69±0.48	0.31±0.53
Choline	3.20(s)	1.04±0.53	1.24±0.70	0.92±0.41	1.13±0.51	1.36±0.63	1.38±0.67	1.34±0.61	1.21±0.49	1.39±0.61	0.90±0.57	0.68±0.48	0.82±0.47
Creatine	3.04(s)	5.03±0.67	6.36±1.74	5.40±1.08	5.18±0.64	6.19±0.74	6.62±0.63	6.67±1.36	5.84±1.20	5.63±0.56	4.59±0.68	4.53±0.47	4.29±0.70
Cytidine	7.81(d)	0.65±0.18	0.47±0.23	0.61±0.45	0.71±0.43	0.67±0.26	0.79±0.34	0.80±0.34	0.93±0.34	0.53±0.08	0.56±0.13	0.60±0.24	0.67±0.18
Dimethylamine	2.72(s)	0.05±0.02	0.05±0.01	0.05±0.02	0.06±0.01	0.02±0.01	0.03±0.01	0.02±0.01	0.02±0.01	0.03±0.02	0.06±0.03	0.05±0.03	0.04±0.02
Ethanolamine	3.86(t)	0.59±0.19	0.73±0.19	0.94±0.25	0.65±0.10	0.58±0.21	0.51±0.14	0.50±0.16	0.53±0.14	0.77±0.20	0.66±0.17	0.52±0.22	0.49±0.16
Formate	8.46(s)	0.53±0.23	0.38±0.15	0.67±0.40	0.26±0.12	0.08±0.05	0.08±0.03	0.11±0.04	0.08±0.02	0.08±0.04	0.06±0.02	0.05±0.10	0.05±0.04
Fumarate	6.52(s)	0.10±0.04	0.10±0.01	0.09±0.04	0.08±0.05	0.11±0.03	0.13±0.03	0.16±0.05	0.12±0.04	0.08±0.01	0.08±0.03	0.06±0.02	0.06±0.02
Glutamate	2.35(m)	6.41±1.27	5.97±1.19	6.57±1.63	7.14±1.01	5.95±1.07	6.36±0.94	5.86±0.85	6.52±0.77	5.13±0.74	3.42±0.58	3.56±1.01	2.95±0.70
Glutamine	2.45(m)	0.61±0.24	0.73±0.16	0.93±0.21	0.70±0.23	0.70±0.07	0.77±0.10	0.79±0.19	0.81±0.16	0.55±0.08	0.43±0.06	0.42±0.04	0.35±0.09
Glycerol	3.66(dd)	0.50±0.34	0.61±0.19	0.92±0.67	0.84±0.30	0.56±0.25	0.58±0.26	0.58±0.20	0.52±0.17	0.57±0.29	0.36±0.22	0.35±0.15	0.33±0.19

Glycine	3.56(s)	0.09±0.14	0.13±0.09	0.24±0.34	0.59±0.47	0.02±0.06	0.04±0.05	0.02±0.05	0.02±0.04	0.10±0.10	0.06±0.05	0.06±0.06	0.06±0.04
Hypoxanthine	8.20(s)	0.65±0.71	0.78±0.70	0.98±0.38	0.52±0.57	0.64±0.27	0.84±0.30	1.07±0.48	0.71±0.29	0.37±0.15	0.47±0.12	0.67±0.65	0.46±0.30
Inosine	8.34(s)	0.56±0.74	0.54±0.66	0.88±0.93	0.68±0.92	1.58±0.73	1.83±0.64	1.75±1.02	1.81±0.91	2.55±0.41	2.01±0.38	1.64±1.28	1.83±0.59
Isobutyrate	1.07(d)	0.03±0.02	0.01±0.01	0.02±0.01	0.02±0.01	0.02±0.02	0.02±0.00	0.02±0.01	0.02±0.01	0.03±0.03	0.03±0.02	0.03±0.01	0.03±0.01
Isoleucine	1.01(d)	1.99±0.56	1.49±0.63	2.48±1.25	2.31±0.56	1.63±0.62	1.87±0.89	2.01±1.01	2.54±1.33	0.94±0.42	1.28±0.44	1.23±0.42	1.35±0.74
Lactate	1.33(d)	13.34±1.23	14.51±2.28	14.52±1.56	12.90±0.63	12.89±2.00	14.37±1.70	14.47±0.93	14.24±1.07	12.46±0.71	15.78±0.86	19.02±1.27	15.14±1.01
Leucine	0.96(t)	3.19±0.92	2.47±1.15	2.76±0.97	3.71±0.94	2.15±0.86	2.58±1.17	2.66±1.41	3.40±1.59	1.36±0.80	1.40±0.62	1.41±0.67	1.71±1.06
Lysine	1.73(m)	1.67±0.97	1.47±0.80	2.34±1.74	2.52±1.41	1.40±0.88	1.89±1.14	1.73±1.23	2.17±1.28	0.82±0.54	0.53±0.47	0.52±0.58	0.53±0.69
Malonate	3.11(s)	0.47±0.14	0.86±0.13	0.66±0.09	0.83±0.24	0.45±0.12	0.67±0.28	0.59±0.35	0.64±0.25	0.36±0.13	0.31±0.09	0.41±0.15	0.28±0.14
Methanol	3.36(s)	0.02±0.03	0.01±0.05	0.02±0.04	0.03±0.03	0.01±0.02	0.03±0.03	0.02±0.04	0.04±0.01	0.02±0.05	0.03±0.02	0.02±0.04	0.03±0.03
Methionine	2.63(t)	0.28±0.08	0.31±0.05	0.26±0.13	0.37±0.14	0.29±0.09	0.32±0.10	0.32±0.13	0.40±0.14	0.14±0.09	0.11±0.04	0.12±0.04	0.15±0.06
myo-Inositol	4.07(m)	0.48±0.37	0.49±0.29	0.36±0.36	0.50±0.24	0.76±0.36	0.70±0.39	0.89±0.41	0.70±0.31	0.73±0.31	0.35±0.39	0.31±0.38	0.31±0.24
Nicotinamide adenine dinucleotide	6.12(d)	0.28±0.34	0.27±0.30	0.42±0.45	0.32±0.42	0.79±0.34	0.90±0.30	0.84±0.52	0.88±0.44	1.22±0.13	1.42±0.18	1.07±0.68	1.13±0.26
Phenylalanine	7.42(s)	0.48±0.11	0.38±0.19	0.48±0.17	0.59±0.12	0.38±0.12	0.45±0.19	0.46±0.23	0.60±0.29	0.20±0.09	0.28±0.09	0.28±0.07	0.31±0.18
Phosphocholine	3.21(s)	1.04±0.53	1.24±0.70	0.92±0.41	1.13±0.51	1.36±0.63	1.38±0.67	1.34±0.61	1.21±0.49	1.39±0.61	0.67±0.57	0.62±0.48	0.53±0.47
Proline	3.33(m)	1.40±0.44	1.45±0.38	1.68±0.58	1.55±0.23	1.21±0.32	1.64±0.31	1.19±0.37	1.37±0.38	0.76±0.47	0.88±0.33	0.75±0.27	0.98±0.35
Pyruvate	2.37(s)	0.89±0.21	0.89±0.18	1.03±0.33	1.03±0.12	0.87±0.21	0.93±0.15	0.81±0.14	0.95±0.13	0.69±0.11	0.85±0.12	0.75±0.18	0.74±0.13
Succinate	2.40(s)	0.04±0.04	0.03±0.03	0.01±0.02	0.04±0.02	0.03±0.03	0.02±0.01	0.01±0.01	0.02±0.01	0.01±0.01	0.02±0.01	0.02±0.01	0.01±0.01
Taurine	3.27(t)	17.80±3.22	31.52±4.51	25.69±7.32	26.27±4.21	18.27±2.64	30.26±4.17	33.94±7.62	30.00±6.88	20.42±2.26	26.87±1.95	26.51±3.51	27.75±3.75
Trimethylamine	2.89(s)	0.01±0.01	0.01±0.01	0.00±0.01	0.01±0.01	0.01±0.00	0.02±0.01	0.02±0.01	0.01±0.01	0.01±0.01	0.01±0.00	0.01±0.01	0.01±0.01
Tyrosine	6.90(d)	1.32±0.30	1.15±0.48	1.68±0.43	1.59±0.32	1.14±0.26	1.28±0.43	1.32±0.51	1.65±0.62	0.71±0.19	0.88±0.22	0.89±0.10	0.93±0.37
Uracil	7.55(d)	0.47±0.15	0.42±0.18	0.42±0.14	0.56±0.20	0.27±0.10	0.31±0.11	0.31±0.08	0.33±0.24	0.13±0.04	0.24±0.09	0.25±0.08	0.25±0.10
Uridine	7.89(d)	0.24±0.23	0.16±0.11	0.38±0.15	0.14±0.17	0.60±0.15	0.67±0.15	0.74±0.17	0.67±0.21	0.67±0.15	0.46±0.12	0.39±0.18	0.43±0.17
Uridine diphosphate glucose	7.96(d)	0.08±0.09	0.11±0.14	0.11±0.16	0.08±0.16	0.01±0.05	0.01±0.05	0.01±0.08	0.04±0.10	0.06±0.11	0.02±0.07	0.01±0.03	0.02±0.04
Urocanate	7.38(s)	0.72±0.15	0.54±0.22	0.64±0.24	0.82±0.13	0.50±0.18	0.61±0.24	0.62±0.29	0.82±0.37	0.31±0.12	0.41±0.13	0.41±0.06	0.42±0.24

Valine	0.99(d)	3.36±0.96	2.69±1.33	4.87±2.37	3.89±0.95	2.76±1.00	3.17±1.25	3.52±1.45	3.88±1.70	1.86±0.71	2.56±0.86	2.39±0.73	2.49±1.07
α-Glucose	5.24(d)	4.04±2.12	3.87±1.49	3.36±2.45	2.90±1.25	3.06±0.97	2.73±0.27	2.53±0.64	2.45±0.58	3.96±0.49	2.49±0.87	2.39±1.38	2.27±0.81
α-Ketoglutarate	3.01(t)	0.91±0.26	0.72±0.22	0.81±0.24	1.40±0.47	0.77±0.14	1.12±0.20	0.97±0.30	1.18±0.45	0.50±0.14	0.43±0.17	0.55±0.12	0.54±0.12
β-Glucose	4.67(d)	2.87±1.54	2.81±1.12	2.61±1.97	2.07±0.79	2.32±0.76	1.96±0.22	1.87±0.46	1.80±0.45	3.08±0.50	1.82±0.73	1.70±1.04	1.75±0.63

SD: the standard deviation
